# *O*-GlcNAcylation: A New Cancer Hallmark?

**DOI:** 10.3389/fendo.2013.00099

**Published:** 2013-08-12

**Authors:** Yann Fardini, Vanessa Dehennaut, Tony Lefebvre, Tarik Issad

**Affiliations:** ^1^Institut Cochin, Université Paris Descartes, CNRS (UMR8104), Paris, France; ^2^INSERM, U1016, Paris, France; ^3^CNRS/UMR 8576, Unit of Structural and Functional Glycobiology, Institut Fédératif de Recherche IFR 147, Lille 1 University, Villeneuve d’Ascq, France

**Keywords:** *O*-glycosylation, *O*-GlcNAc, post-translational modification, cancer, metastasis, cell cycle, epigenetics, transcription factors

## Abstract

O-linked *N*-acetylglucosaminylation (*O*-GlcNAcylation) is a reversible post-translational modification consisting in the addition of a sugar moiety to serine/threonine residues of cytosolic or nuclear proteins. Catalyzed by *O*-GlcNAc-transferase (OGT) and removed by *O*-GlcNAcase, this dynamic modification is dependent on environmental glucose concentration. *O*-GlcNAcylation regulates the activities of a wide panel of proteins involved in almost all aspects of cell biology. As a nutrient sensor, *O*-GlcNAcylation can relay the effects of excessive nutritional intake, an important cancer risk factor, on protein activities and cellular functions. Indeed, *O*-GlcNAcylation has been shown to play a significant role in cancer development through different mechanisms. *O*-GlcNAcylation and OGT levels are increased in different cancers (breast, prostate, colon…) and vary during cell cycle progression. Modulating their expression or activity can alter cancer cell proliferation and/or invasion. Interestingly, major oncogenic factors have been shown to be directly *O*-GlcNAcylated (p53, MYC, NFκB, β-catenin…). Furthermore, chromatin dynamics is modulated by *O*-GlcNAc. DNA methylation enzymes of the Tet family, involved epigenetic alterations associated with cancer, were recently found to interact with and target OGT to multi-molecular chromatin-remodeling complexes. Consistently, histones are subjected to *O*-GlcNAc modifications which regulate their function. Increasing number of evidences point out the central involvement of *O*-GlcNAcylation in tumorigenesis, justifying the attention received as a potential new approach for cancer treatment. However, comprehension of the underlying mechanism remains at its beginnings. Future challenge will be to address directly the role of *O*-GlcNAc-modified residues in oncogenic-related proteins to eventually propose novel strategies to alter cancer development and/or progression.

## Introduction

Excess food intake associated with modern lifestyle constitutes an important cancer risk factor (1). Numerous epidemiological studies indicate that obesity or diabetic conditions increase the risk of cancer, including colon, esophageal, liver, pancreas kidney, endometrial, and breast cancers (2, 3). Increased body mass index (BMI) above 25 kg/m^2^ is associated with a significantly increased relative risk of several cancers, and BMI higher than 30 kg/m^2^ are associated with a two- to four-fold increase in colorectal, endometrial, esophageal, liver, gallbladder, and gastric cancers (4). Hyperglycemia also appears to be an important cancer risk factor. Indeed, in a 10-year prospective study involving 1,298 million Koreans, individuals with fasting serum glucose higher than 140 mg/dl had significant higher death rates from all cancers combined than those with fasting glucose lower than 90 mg/dl. Sustained weight loss, improvement of insulin resistance, and attenuation of metabolic syndrome observed after bariatric surgery are associated with reduction in cancer incidence (5). In mouse and rat models of diet-induced obesity, overfeeding is associated with accelerated development of tumors (6). In contrast, food restriction has inhibitory effects on tumor growth in rodents (7) and reduces cancer incidence in non-human primates (8). Interestingly, low calorie intake habits in Okinawan population (9) is associated with reduced cancer risk compared to mainland Japan (10), suggesting that the anti-cancer effects of calorie restriction in rodent models may extend to primates, including humans.

Nutritional conditions, excess body weight, and insulin resistance can modulate tumor development by modifying circulating factors that affect signaling pathways involved in cell growth, proliferation, and apoptosis. For instance, chronic hyperinsulinemia is associated with increased risk of colon, pancreas, breast, and endometrium cancers (4). These effects could be directly mediated by insulin receptors on (pre)neoplastic target cells, or might be secondary to hyperinsulinemia. Thus, insulin promotes the synthesis of IGF1, and inhibits the expression of insulin-like growth factor binding proteins 1 and 2 (IGFBP1 and IGFBP2), resulting in increased bioavailability of this potent growth factor. In addition, insulin and IGF1 inhibit the expression of sex-hormone binding globulin (SHBG), resulting in increase in estrogen bioavailability, a breast cancer risk factor in post-menopausal women. Increased leptin circulating levels associated with excess adiposity may also constitute a risk factor for breast cancer. Indeed, ObRb, the long form of the leptin receptor stimulates proliferative and anti-apoptotic pathways, and both leptin and leptin receptors are overexpressed in human primary and metastatic breast cancer cells (11, 12).

**Figure 1:**
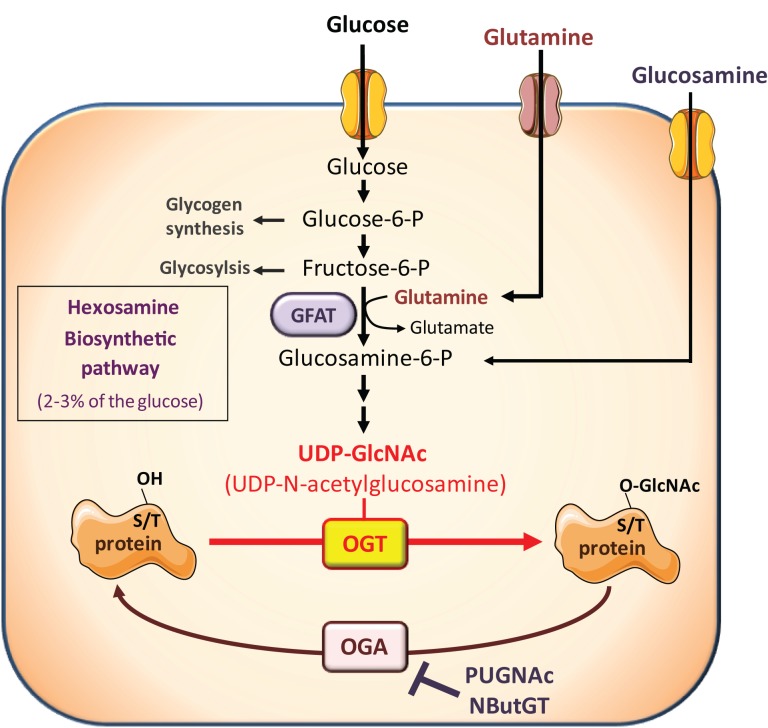
**The hexosamine biosynthetic pathway and protein *O*-GlcNAcylation**. The hexosamine biosynthetic pathway (HBP) controls *O*-GlcNAc glycosylation (*O*-GlcNAcylation) of nuclear and cytosolic proteins. This dynamic and reversible post-translational modification controls activity, localization, or stability of substrate proteins, according to the rate of glucose availability. A fraction (2–3%) of glucose entering the cell is directed to the HBP. In this pathway, fructose-6-phosphate is converted to glucosamine-6-phosphate by the glutamine:fructose-6-phosphate amidotransferase (GFAT), the rate-limiting enzyme of the pathway. After a subset of reactions, UDP-*N*-acetylglucosamine (UDP-GlcNAc) is generated and used by the *O*-GlcNAc-transferase (OGT) as a substrate to add GlcNAc to serine or threonine residues of target proteins. *O*-GlcNAc moiety is removed from *O*-GlcNAc-modified proteins by the *O*-GlcNAcase (OGA). Experimentally, the level of *O*-GlcNAc-modified proteins in cells can be manipulated by exposing cells to high glucose concentrations or to glucosamine which enters the HBP downstream the rate-limiting GFAT-mediated reaction, as glucosamine-6-phosphate. In addition, OGA can be inhibited by pharmacological agents such as *O*-[2-acetamid-*O*-2-deoxy-D-glucopyranosylidene] amino-*N*-phenylcarbamate (PUGNAc) or 1,2-dideoxy-2′-propyl-alpha-d-glucopyranoso-[2,1-D]-Delta 2′-thiazoline (NButGT), resulting in an accumulation of *O*-GlcNAc-modified proteins in the cell.

However, besides its effects on circulating hormones and adipokines, nutritional conditions also modulate the availability of nutrients important for growth and proliferation of cancer cells. Among them, glucose and glutamine are considered crucial, and cancer cells have been described as “addicted” to these two nutrients, from which they obtain biosynthetic precursors to build cell membranes, DNA, and proteins. Glucose and glutamine are the two most abundant extracellular nutrients (13). They contribute carbons to the synthesis of the three major classes of macromolecules (nucleic acids, lipids, and proteins) in proliferating tumor cells. In addition to its role as a carbon source, glutamine also donates nitrogen to nucleotide and amino acid synthesis. Thus, biosynthesis of nucleotides utilizes ribose 5-phophate produced from the diversion of glycolytic intermediate into the pentose phosphate pathway, and glutamine. Fatty acid synthesis, used to produce lipids, requires acetylCoA generated from glucose. Protein synthesis requires amino acids, tRNAs, and ribosomes. Both glucose and glutamine are used to generate these molecules (13). However, in addition to their role as “molecular bricks” in building of cancer cell components, glucose and glutamine metabolism intervene in protein *O*-GlcNAcylation, a post-translational modification that regulates most aspects of cell life (14–18). *O*-GlcNAcylation of cytosolic and nuclear proteins is a reversible post-translational modification, analogous to phosphorylation, which controls protein subcellular localization, stability, or activity according to the nutritional environment. It corresponds to the addition of *N*-acetylglucosamine (GlcNAc) on serine or threonine residues. The reaction is catalyzed by *O*-GlcNAc-transferase (OGT), which uses UDP-GlcNAc as a substrate (Figure 1). UDP-GlcNAc, produced through the hexosamine biosynthetic pathway (HBP), can be considered as a sensor for the nutritional state of the cell, as it integrates glucose, glutamine, fatty acids (acetyl), uridine, and ATP metabolism (14–18). *O*-GlcNAc is removed from proteins by *O*-GlcNAcase (OGA), permitting dynamic regulation of *O*-GlcNAcylation levels. *O*-GlcNAcylation can be in competition directly or indirectly with phosphorylation on the same protein, providing a complex cross-talk between these two PTM to control the function of various proteins (19).

Excess of nutrients intake, hyperglycemia, and other metabolic perturbations associated with diabetes and obesity are believed to feed the HBP and promote abnormally elevated *O*-GlcNAcylation of key signaling molecules and transcription factors. These modifications have been proposed to play key roles in complications associated with the metabolic syndrome, diabetic conditions, neurodegenerative disease, and cancer (17, 20, 21).

On the other hand, *O*-GlcNAcylation regulates cell cycle, signaling intermediates, and transcription factors involved in the control of cell proliferation or cell death (22–27). A growing amount of studies indicates that *O*-GlcNAcylation may constitute an important regulator of cancer growth and invasion, providing a potential link between obesity, diabetes, and cancer (28, 29).

## *O*-GlcNAcylation and *O*-GlcNAc-Cycling Enzymes in Cancer

Increased protein *O*-GlcNAcylation and changes in OGT and/or OGA expression have now been described in different cancer types including breast (30–32), lung (33), colon (33), liver (34), bladder (35), endometrial (36) prostate (37), and chronic lymphocytic leukemia (CCL) cells (38).

### Breast cancer

Breast cancer is the most common cancer in women. The link between nutritional conditions, obesity, and breast cancer is well established (39). Initial studies by Slawson et al. suggested increased OGA activity in primary breast tumors compared to matched adjacent breast tissues (30). However, because the data set was relatively small, no clear correlation could be established with tumor grade or type. Moreover, these results contrasted with those obtained by other groups in more recent investigations. Dahl et al. defined a set of 40 candidate genes that are predominantly localized in the most frequently altered chromosomal regions known to be important in the pathogenesis of breast and ovarian cancers (40). Systematic characterization of these candidate genes by both cDNA dot plot using cancer profiling array and real-time RT-PCR analysis revealed differential expression in breast cancer for nine genes, including *MGEA5*, the gene coding for OGA, which expression was reduced by about 56% in breast tumors (40). In agreement with decreased OGA in breast cancer, Gu et al. using immunohistochemistry analysis, observed that the global *O*-GlcNAcylation level in breast tumor tissues was significantly elevated compared to the corresponding adjacent normal tissue (31). Moreover, *O*-GlcNAcylation was also significantly enhanced in metastatic lymph nodes compared with their corresponding primary breast tissues, suggesting a potential influence of *O*-GlcNAcylation on malignant properties of the breast cancer cells (31). More recently, Krzeslak et al. observed increased OGT and decreased OGA mRNA expression in breast tumors (32), with poorly differentiated tumors (grade II and III) having significantly higher OGT and lower OGA mRNA expression than grade I tumors, respectively. Lymph node metastasis status was also significantly associated with decreased OGA mRNA expression, but not with OGT mRNA expression. OGT and OGA expression profiles showed no significant differences in association with different estrogen and progesterone status (32). These results have been confirmed very recently by Champattanachai and colleagues who analyzed breast tumors from grade I to III and observed increased total *O*-GlcNAc protein levels along with OGT levels compared to healthy tissue. This increase was also proportional to tumor grades (41).

Finally, a role for *O*-GlcNAcylation in tamoxifen sensitivity was recently described (42). In breast cancer, tamoxifen remains largely used as endocrine therapy, but resistance to this drug often occurs (43). Kanwal et al. observed that *O*-GlcNAcylation-inducing treatment protected MCF-7 cells from tamoxifen-induced cell death, whereas siRNA-mediated OGT inhibition had opposite effects. These data suggest that targeting OGT might be an interesting approach to overcome tamoxifen resistance in breast cancer (42).

### Colorectal cancer

Colorectal cancers (CRCs) are one of the leading causes of mortality and morbidity by cancer, the second for women and the third for men, in Europe and the United States of America. Unhealthy lifestyle and nutrient excess favor emergence of CRCs and, more generally, these cancers are intimately linked to metabolic diseases that themselves reach epidemic proportions in the Western societies. As an example, type-2 diabetes doubles CRC emergence (44). Evaluation of *O*-GlcNAc in CRC using immunohistochemical analyses revealed that *O*-GlcNAcylation level and OGT expression are increased in colon tissues in comparison with the corresponding adjacent tissues, whereas no significant difference was observed for OGA (33).

### Liver cancer

Although liver cancers (LCs) only represent the fifth and the seventh most common cause of cancer in men and women respectively, their frequency has been increasing steadily for 20 years. Indeed, as for CRCs, unhealthy lifestyle favors emergence of LCs, and diabetes and obesity increase the risk of LCs through the development of a non-alcoholic steatosis (45).

Zhu et al. (34) analyzed *O*-GlcNAcylation level and expression of OGT and OGA by real-time PCR in tissues of patients suffering hepatocellular carcinoma, the most common primary liver malignancy (600,000 new cases diagnosed each year). Immunohistochemistry analyses of patients having undergone a liver transplantation, that remains at the present time the best treatment for LC and cirrhosis despite the high incidence of tumor recurrence, indicate that *O*-GlcNAcylation was higher in cancer tissues compared to healthy tissues. Moreover, *O*-GlcNAcylation was higher again in patients diagnosed a recurrence of hepatocarcinoma. The authors tried to correlate OGT and OGA expression with the prognosis of patients who underwent liver transplantation. The 60 tumors used were segregated into two groups according to low/high amounts of OGT/OGA transcripts. More than three quarters of the recurrent tumor tissues show low OGA expression while the non-recurrent ones have high OGA levels. Intriguingly, no significant correlation for OGT was found. The recurrence-free survival was significantly better for patients showing high OGA than patients with low OGA, while OGT expression did not allow predicting the prognosis. The authors proposed that low expression of OGA may constitute an additional independent prognostic factor for the recurrence-free survival following liver transplantation.

### Bladder and endometrial cancers

Krzeslak et al. measured the level of OGA and OGT mRNA in bladder (35) and endometrial (36) cancers. Rozanski et al. observed that OGA mRNA was present in the urine (cells pelleted from centrifuged urines) of healthy and bladder cancer patients with almost the same proportion (47.1% of healthy and 52.3% of bladder cancers patients). OGT mRNA was found in 50% of patients suffering bladder cancer while it was not detected in healthy individuals. OGA mRNA level was higher in grade I tumors compared to grade III, whereas OGT mRNA was lower in grade I than in grades II and III. The authors concluded that measurement of OGT and OGA mRNA in urine might be an interesting parameter for the diagnosis bladder cancers (35).

Regarding the endometrial carcinomas, it appeared that OGT and OGA mRNA were significantly more elevated in grades II and III tumors than in grade I. In addition, OGT and OGA expression was higher in case of cancers with deep myometrial invasion. Although both OGT and OGA mRNA were increased, the relative expression of OGT was much higher than OGA. However, no significant differences in enzymes messengers were found for the different lymph nodes metastasis (36).

### Other cancers

Changes in *O*-GlcNAc levels and/or expression of *O*-GlcNAc-cycling enzymes have also been described in leukemia, lung, and prostate cancers (33, 37, 38).

Increased protein *O*-GlcNAcylation and OGT expression were observed in CCL cells compared to normal circulating and tonsillar B cells (38). However, higher *O*-GlcNAc levels in CCL cells were associated with a favorable outcome, while lower levels were associated with more aggressive disease.

Mi et al. constructed a tissue microarray comprising 31 paraffin-embedded lung cancer tissues and their corresponding adjacent normal lung tissues (33). Using immunohistochemistry, the authors observed increased *O*-GlcNAcylation level and OGT expression in cancer tissues, whereas OGA expression level was not modified. In agreement with their results, examining Oncomine™ database, Mi et al. found increased OGT mRNA expression in lung cancers (33).

Lynch et al. (37) analyzed Oncomine™ database to determine whether OGT was overexpressed in tumors, and found four microarray gene expression studies showing elevated OGT mRNA levels in human prostate carcinoma as compared to adjacent tissue samples. In addition, a survey of the National Center for Biotechnology Information Gene Expression Omnibus indicated a positive correlation between high OGT expression and metastatic progression between normal, primary tumor, and metastatic tumor tissues. Moreover, an additional study of 94 patient tumor samples, which when stratified by level of OGT expression, indicated that disease-free survival 5 years post-treatment was higher in patients with low OGT expression profile compared to patients with increased OGT expression. In agreement with these data, using a panel of normal and prostate carcinoma cell lines, Lynch et al. observed an increased *O*-GlcNAc level and OGT expression associated with malignant properties in prostate cancer cells (37).

In summary, studies on different types of cancer generally indicate increased expression in *O*-GlcNAc level and OGT expression. However, contradictory results were obtained concerning OGA, with either decreased (31, 32, 34, 35, 40), no change (33), and even increased in OGA levels in some studies (30, 36, 46). These discrepancies in OGA results may originate from the fact that OGA expression itself is upregulated upon increase *O*-GlcNAcylation level in the cell (47), complicating the interpretation of the data.

From the literature reviewed in the previous sections, it appears that cancer cells display both increased uptake of nutriments involved in *O*-GlcNAc biosynthesis and increased capacity to *O*-GlcNAcylate proteins. As we shall see in the following sections, alteration in *O*-GlcNAcylation may directly affect important steps in tumorigenesis.

## *O*-GlcNAcylation and Mitogenic Signaling Pathways

As mentioned previously, the nutritional status impacts tumor development. Thus, several studies in rodent showed that dietary restriction can inhibit tumor growth. Mitogenic signals elicited by various receptors often involve common signaling cascades such as PI3K/Akt or MAPK pathways. Interestingly, when grown as tumor xenografts in mice, cancer cells bearing mutations that induce constitutive PI3K activation are resistant to dietary restriction, whereas cancer cells with mutations that constitutively activate the Ras/Raf/MAPK pathway remain sensitive to calorie restriction (48, 49). These observations suggest that the activity of the PI3K/Akt pathway is central to sensitivity of cancer cells to nutritional conditions. Interestingly, *O*-GlcNAcylation has been implicated in modulation of PI3K/Akt activity. For instance, in thyroid anaplastic cancer 8305C cells, increased *O*-GlcNAcylation induced by OGA inhibition resulted in an increase in basal and IGF1 stimulated Akt activity (32). In MCF-7 breast cancer cells, Olivier-Van Stichelen et al. showed that serum-induced Akt activation was markedly impaired by siRNA-mediated OGT inhibition (27). Moreover, Kanwal et al. showed that treating MCF-7 cells with *O*-GlcNAcylation-inducing agents stimulated Akt phosphorylation (42). Interestingly, using a BRET-based assay which monitors PIP3 production at the plasma membrane in living cells (50), Kanwal et al. demonstrated that *O*-GlcNAcylation-inducing treatments stimulated the production of phosphatidylinositol 3,4,5-trisphosphate (PIP3) by PI3K in MCF-7 cells, suggesting that stimulation of Akt by these treatments resulted from activation of early steps in the signaling cascade (42). Several anti-cancer drugs targeting PI3K are under clinical development (51). Noteworthy, in a cancer cell line collection (60 diverse human cancer cell lines representing multiple tumor types), resistance to treatment with the PI3K inhibitor GDC-0941 correlated with OGT expression level (52). These authors also showed in different cell lines that silencing OGT increased sensitivity to GDC-0941, whereas increasing *O*-GlcNAc levels using PUGNAc promoted resistance to this drug (52).

On the other hand, growth factor signaling may also influence the *O*-GlcNAcylation pathway. For instance, it has been shown that upon insulin stimulation, OGT is recruited to the plasma membrane through a PIP3-binding domain, leading to *O*-GlcNAcylation and inhibition of insulin signaling intermediates (53). This is consistent with results from another study showing that insulin stimulation increased OGT shuttling from nucleus to cytoplasm. This was associated with OGT interaction with IR, allowing the latter to phosphorylate OGT on tyrosine residues and stimulate its catalytic activity (54). Very recently, OGT recruitment in response to insulin was shown to occur in lipid rafts through a PI3K/Akt dependent pathway in HepG2 cells. Notably, expression of OGT is required for proper IR expression and insulin signaling in HepG2 cells, illustrating the complex interconnections between these two pathways (55).

Growth factors can also affect *O*-GlcNAc signaling by regulating the expression of important enzymes of pathway. For instance, one of the first links between mitogenic signals and hexosamine pathway was provided by the observation that in MDA-468 breast cancer cells, epidermal growth factor activated the expression of the rate-limiting enzyme GFAT (56). More recently, serum stimulation was shown to increase OGT protein levels in MCF-7 cells through a post-transcriptional dependent pathway, and this effect appears to be necessary for cell growth (27). However, besides these specific examples, no systematic approaches on growth factors or hormones in relation to their ability to control *O*-GlcNAcylation and/or *O*-GlcNAc-cycling enzyme expression have been conducted. Clearly, the relevance of these mechanisms in breast cancer pathophysiology still deserves further investigations.

## *O*-GlcNAcylation and Cell Cycle Regulation

### The first observations

Independent reports conducted during the 1990s supported that OGT and *O*-GlcNAc dynamics interfere with cell cycle progression of germinal and somatic cells.

It was first highlighted dynamic *O*-GlcNAcylation changes in response to mitogens (57). Activation of murine T lymphocytes with the lectin concanavalin A resulted in a rapid decrease in cytosolic proteins *O*-GlcNAc level and, conversely, an enhancement of *O*-GlcNAcylation of nuclear proteins. These observations suggested that *O*-GlcNAc cycling was necessary for the activation of T cells. In the human colon adenocarcinoma cell line HT29, colcemid or nocodazole-induced G2/M cell cycle arrest induced a concomitant enhancement in phosphorylation and *O*-GlcNAcylation of keratins K8 and K18 (two intermediate filament proteins overexpressed in cancer cells) (58). Removal of nocodazole slowly returned keratins *O*-GlcNAc to baseline levels, suggesting that *O*-GlcNAcylation of these proteins is cell cycle dependent. At this time, because of the lack of techniques allowing specific visualization of *O*-GlcNAcylated proteins, the impact of *O*-GlcNAcylation on cell cycle progression was largely minimized. Since then, it appeared that *O*-GlcNAcylation actively contributes in cell cycle proceeding at different steps. For instance, inhibition of OGA using the non-selective hexosaminidase inhibitor PUGNAc indicated that treated-cells progressed more slowly through the cell cycle than untreated ones (47).

### G0/G1

**Figure 2:**
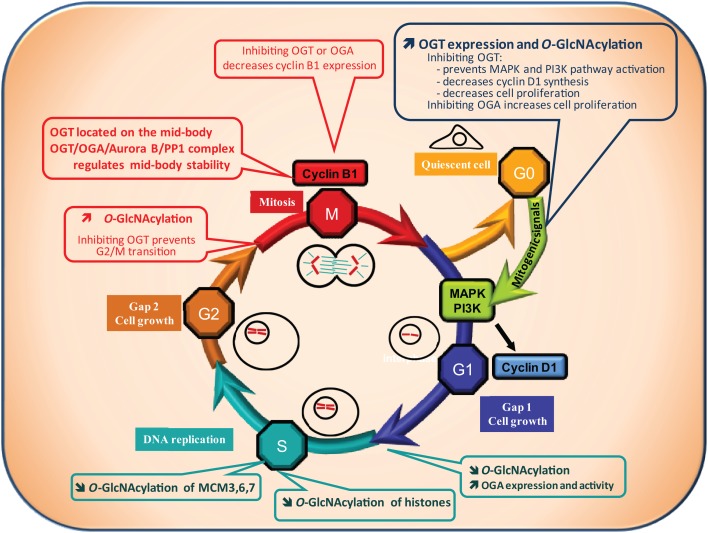
***O*-GlcNAcylation and cell cycle**. A quiescent cell enters the cell cycle upon mitogenic signals. Cell cycle is divided into four phases: the G1 (Gap1) phase, during which cell grows, followed by the S phase of DNA replication, then the G2 (Gap2) phase which prepares the cell for the proper division phase called M phase. Progression of the cell through the different phases is highly controlled: at the G1/S and G2/M transitions a checkpoint exists to ensure that DNA is not damaged respectively before and after its replication. The G2/M checkpoint also controls that replication is ended before division of the cell into two daughter cells genetically identical. *O*-GlcNAcylation levels have been found to vary all along the cell cycle suggesting that it could regulate this process. OGT and *O*-GlcNAcylation levels increase when quiescent cells are stimulated by mitogenic signals to enter into the cell cycle (G0/G1 transition). On the contrary, OGA activity is increased at the G1/S transition leading to a decrease in global *O*-GlcNAcylation levels. At the G2/M checkpoint, a burst in *O*-GlcNAcylation occurs. In agreement with these observations, *O*-GlcNAcylation has been demonstrated to be crucial for cell cycle entry and progress. Inhibition of OGT delays serum-stimulated MAPK and PI3K pathways activation and cell cycle entry whereas OGA inhibition accelerates the process. Moreover, inhibiting OGT in G2-like *Xenopus laevis* oocytes prevents entry into M phase. In addition, the levels of cyclin D1 and cyclin B1, two key regulators of the G1 and the M phase respectively, decrease when OGT is inhibited. Finally, *O*-GlcNAcylation could take part in the control of DNA replication since it modifies several histones and three proteins of the MCM (Mini Chromosome Maintenance Complex) 3, 6, and 7 that belong to the DNA pre-replication complex. As a consequence, deregulation of *O*-GlcNAcylation processes could contribute to perturbation in cell cycle control leading to anarchic proliferation, but also in accumulation of DNA mutations, two well established characteristics of cancer cells.

Most of the cells of the organism are in the G0 phase, a quiescent stage of non-division. Entry into G1 phase (the first phase of the cell cycle) requires the presence of exogenous mitogenic signals that lead to activation of the MAP kinase and PI3K pathways, and to transcription of the cyclin D1 gene, a key regulator of the G1 phase (Figure 2).

Recently, two independent groups reported that OGT is significantly increased following serum stimulation (G0/G1 transition) (59, 60). Blockade of OGT activity or interfering with its expression delays serum-stimulated cyclin D1 synthesis and cell proliferation (27). Kwei et al. observed also in the ovarian cancer cell line OVCAR-4 that cyclin D1 is down-regulated in siOGT-transfected cells (52). A decrease in the HBP flux also reduces the cell proliferating rate, while inhibiting OGA accelerates this process. OGT silencing diminishes PI3K and MAPK activation (27), demonstrating that OGT is indispensable for G0/G1 transition.

Therefore, studies that focused on G0/G1 transition indicate that OGT synthesis and a subsequent *O*-GlcNAcylation increase are required for cell cycle entry. It might be interesting to establish whether cyclin D is itself *O*-GlcNAcylated, and to determine its potential consequences on its expression and activity.

### G1/S

The S phase is the second phase of the cell cycle during which DNA replicates. At the G1/S boundary a checkpoint is set up to make sure that the DNA is not damaged. OGT, OGA, and *O*-GlcNAcylation levels were measured during the G1/S transition and throughout S-phase progression (59). A global decrease in *O*-GlcNAcylated proteins was observed during the G1/S transition and this change correlates with an increase in OGA expression and activity. In agreement with this observation, inhibition of HBP accelerated the S phase (47). Drougat et al. identified more than 50 proteins which *O*-GlcNAc status varies among which Minichromosome Maintenance (MCM) 3, 6, and 7, which are localized in the multiprotein pre-replicative complex needed for helicase activity and are involved in the replication of DNA (59). Zhang et al. showed that *O*-GlcNAcylation of histones is lower in S phase in comparison with G1, late S, and G2 phases (61) (see below for details). Accordingly, reduction of histones *O*-GlcNAcylation during S phase may help the pre-replicative complex to reach the chromatin.

Therefore, the few studies relating to the impact of *O*-GlcNAcylation on G1/S transition indicate that contrary to G0/G1, a global decrease in *O*-GlcNAcylation is observed. Intriguingly, not only OGA but also OGT levels increase during G1/S (59). It is tempting to hypothesize that OGT plays a function independent of its GlcNAc transferase activity as exemplified by its interaction with p38MAPK (62).

### G2/M

The S phase is followed by G2, a phase during which the cell grows and prepares for the proper division M phase (mitosis in the case of somatic cells and meiosis of germ cells). A second checkpoint exists at the G2/M transition to ensure that the DNA replication is ended and that the DNA is not damaged.

The *Xenopus laevis* oocyte has been widely used as a model for the characterization and the identification of many key-cell cycle components, such as the M-phase promoting factor (MPF) and the cytostatic factor (CSF) (63, 64) and for studying the regulation of the cell cycle, especially events occurring at G2/M. At the end of oogenesis, the oocyte is physiologically blocked in prophase of the first meiotic division in a G2-like stage; it is called immature oocyte. Upon progesterone stimulation, the oocyte resumes meiosis in a G2/M analog transition phase.

The micro-injection into *Xenopus* oocytes of bovine galactosyltransferase, an enzyme enabling the capping of terminal GlcNAc residues inhibited M-phase entry and blocked M to S-phase transition (65). Slawson and co-workers showed that the perturbation of *Xenopus* oocyte *O*-GlcNAcylation levels either by glucosamine or PUGNAc treatment modified the maturation kinetics (66). A few years later, a set of studies conducted by the same group showed that hormonal stimulation of physiologically G2-blocked *Xenopus laevis* oocytes triggers a quick increase in *O*-GlcNAcylation levels and that inhibition of OGT impairs G2/M transition (67–70). OGT and *O*-GlcNAc localized on the meiotic spindle and chromosomes in metaphase-II *Xenopus laevis* oocytes. It was also observed that OGT expression and *O*-GlcNAcylation peaked at the M phase of the cell cycle of MEF and HEK293 cells (60). Slawson et al. observed that OGT localized to the mitotic spindle and midbody during mitosis and that its overexpression resulted in supernumerary chromosomes (47). In a second report, these authors showed that OGT and OGA interact with Aurora B and protein phosphatase 1 (PP1) to regulate the stability of the midbody and the phosphorylation and/or *O*-GlcNAcylation of vimentin at M phase (71). Regarding G2/M transition, both OGA and OGT knockdown decrease cyclin B1 expression, indicating that a correct expression of the two enzymes are necessary for cell cycle progression (27, 60).

As for cell cycle entry, *O*-GlcNAcylation increase is crucial for the cell to start mitosis. The cell cycle is a very complex process that requires a plethora of enzymes and regulatory components. These proteins were widely described to be regulated by PTM like phosphorylation, acetylation, methylation, ubiquitination, and many others. Recently, *O*-GlcNAcylation has joined this list of PTM, and manipulating OGT and OGA level or activity affects the progression of the cell cycle.

Altogether, the available data indicate that *O*-GlcNAcylation tends to increase all along the cycle with a drop at G1/S. The pattern of *O*-GlcNAcylation levels after the M phase and during cell cycle exit remains unknown. One could expect a dramatic decrease, a hypothesis that needs to be experimentally addressed.

## *O*-GlcNAcylation and Epigenetic Regulations

Recent whole-exon sequencing of human cancers has shown a high proportion of mutations in genes involved in regulation of DNA methylation, histone modification, and/or nucleosome remodeling (72). These discoveries firmly establish that interferences in epigenetic processes can lead to cancer and add credence to the idea that epigenetics is a major player in this disease. As described below, it is now clear that *O*-GlcNAcylation plays a part in the regulation of the epigenome.

Over the last 4 years, several groups focused on *O*-GlcNAc-mediated regulation of chromatin dynamics, a process crucial for DNA replication, DNA repair, gene expression, and mitosis (DNA compaction) (33, 73–75).

Chromatin condensation and relaxation is managed by histones that form octamers by assembling two copies of each core nucleosomal histone, H2A, H2B, H3, and H4. These oligomers interact with DNA in a nucleosome structure compacted by the linker histone H1. Histones, assisted by numerous post-translational modifications are therefore the main proteins responsible for chromatin remodeling and gene expression. Acetylation, methylation, and phosphorylation are the best-characterized histones PTM, the two formers being associated with activation of chromatin and the latter with both activation and repression [for review, see (76)]. Histones are also covalently modified by ADP-Ribosylation, SUMOylation, ubiquitination, and as recently described, *O*-GlcNAcylation [for review, see (77)], while *O*-GlcNAcylation of *Xenopus* oocytes histones remained undetectable (69). The histone code is therefore very complex and far from being deciphered.

Sakabe and Hart pointed out the impact of OGT on histone H3 modification (74). These authors mapped three *O*-GlcNAcylation sites on H2A, H2B, and H4 at Thr101, Ser36, and Ser47 respectively, three phosphorylation sites necessary for the assembly of nucleosomes (74). H2B tail is also *O*-GlcNAcylated at Ser112 (78). This modification promotes H2B monoubiquitination at Lys120 allowing local transcription as suggested by visualization of *O*-GlcNAcylated H2B at Ser112 in transcribed gene loci (78). H3 tail is *O*-GlcNAcylated at the phosphorylation site Ser10 (61). Histone *O*-GlcNAcylation is coupled with other modifications associated with both active and inactive chromatin states, and covalent linkage of histones by *O*-GlcNAc fluctuates all along the cell cycle with a lower rate in S phase (61). Fong et al. worked on the same histone, and identified Thr32 as a major H3 *O*-GlcNAcylated site (75). *O*-GlcNAcylated H3 isoforms are higher in interphase cells than in mitosis. H3 phosphorylation at Ser10, Ser28, and Thr32 is associated to mitosis: increased H3 *O*-GlcNAcylation reduces phosphorylation and delays mitosis entry (75). It is noteworthy that H3 Ser10 and Ser28 are phosphorylated by Aurora B (79, 80) and dephosphorylated by PP1 (81), two enzymes physically interacting with OGT and OGA (71). It is tempting to hypothesize that regulation of histone assembly/disassembly is managed in part by a heterotetrameric complex made of Aurora B, PP1, OGT, and OGA.

*O*-GlcNAc-transferase and *O*-GlcNAc may also affect chromatin structure by modulating chromatin-remodeling enzyme activities. Fujiki et al. (82) highlighted the role *O*-GlcNAcylation of a histone lysine methyltransferase (MLL5) in the control of chromatin state. MLL5 *O*-activates RAR-alpha and interacts with OGT in an active multimeric complex. *O*-GlcNAcylation of MLL5 increases granulopoiesis of HL60 promyelocytes in response to retinoic acid. This facilitation occurs through an increased-MLL5 methylation induced by *O*-GlcNAcylation. One *O*-GlcNAcylation site was identified on MLL5 at Thr440 and modification of this residue potentiates its H3K4 methyltransferase activity. Another study by Sakabe and Hart also observed that moderate OGT overexpression prevented the phosphorylation of coactivator-associated arginine methyltransferase 1 (CARM1), resulting in decreased H3 methylation on Arg17 by CARM1 (74).

TET (ten-eleven translocation) proteins interact with and target OGT to chromatin (83). TET are DNA hydroxylases involved in DNA demethylation. These enzymes convert 5-methyl-Cytosine to 5-formyl-Cytosine and 5-carboxyl-Cytosine successively (84). TET are necessary for gene transcription, pre-mRNA splicing, zygotic epigenetic reprograming, and TET mutations are responsible for myeloid cancers. In an attempt to identify TET proteins partners, Chen et al. (85) showed that OGT interacts with TET2 and TET3. While OGT does not influence TET function, physical interaction between TET2 and OGT is a precondition to address the glycosyltransferase to chromatin where it *O*-GlcNAcylates histones. Knockdown of TET2 decreases OGT interaction with chromatin and impairs H2A/B, H3, and H4 *O*-GlcNAcylation, and deregulation of TET2 more precisely reduces *O*-GlcNAcylation of H2B at Ser112. Genome-wide chromatin immunoprecipitation and sequencing analysis indicate that OGT and H2B Ser112 *O*-GlcNAc overlap a large amount of target genes with TET2 and that the density of distribution is enriched at transcription start sites. As *O*-GlcNAcylation of H2B at Ser112 promotes monoubiquitination and transcriptional activation (78), TET2 may exert chromatin activation and gene expression by promoting demethylation of DNA and nucleosome opening by acting indirectly on histones. Therefore, TET2 and OGT form a complex that regulates gene transcription. Another study reports the interaction of TET1, TET2, Sin3a, and Hcfc1 with nuclear OGT (86). A genome-wide range analysis led by chromatin immunoprecipitation coupled to high-throughput DNA sequencing indicates that 62% of the 11552 OGT binding sites locate within promoter regions. Among the list of genes regulated by OGT, it was found genes involved in glycerolipid, glycerophospholipid, N- and *O*-glycosylation metabolism. OGT binding sites overlay with TET1 at H3K4me3 positive promoters, and both proteins interact together in the vicinity of transcription start sites enriched in unmethylated CpG-rich promoters. As for TET2, TET1 is necessary for recruiting OGT to the chromatin but it seems that, contrary to TET2 and TET3 that are neither *O*-GlcNAcylated nor regulated by OGT (85, 87), OGT regulates 5-hydroxymethyl-Cytosine levels by stabilizing TET1 at the promoters.

## *O*-GlcNAcylation Regulates Transcription Factor Activities

**Figure 3:**
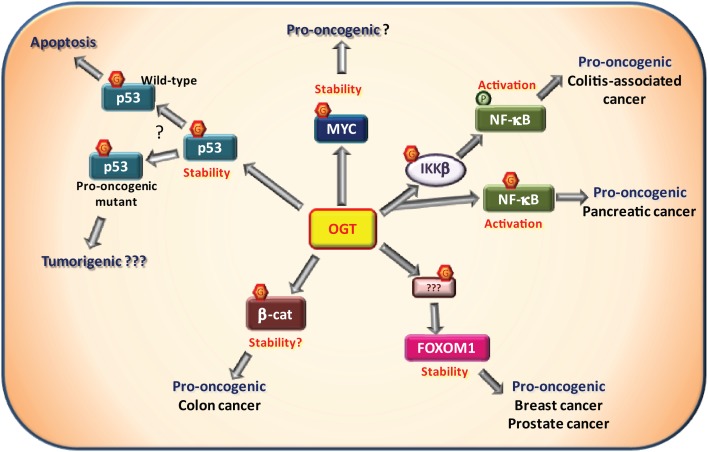
***O*-GlcNAcylation and oncogenic transcription factors**. Several transcription factors have been described to be *O*-GlcNAcylated by the *O*-GlcNAc-transferase (OGT) among which some that are involved in tumorigenesis. The proto-oncogene MYC has been shown to be *O*-GlcNAcylated on Thr58. This site is normally phosphorylated by GSK3β in response to MYC phosphorylation on Ser62 resulting in MYC degradation. Proper experimental evidences demonstrating that *O*-GlcNAcylation promotes MYC stability and pro-oncogenic activities remain to be described. NF-κB signaling is known to be promoted by *O*-GlcNAcylation. First, the IKKβ kinase responsible for NF-κB activation has been shown to be to *O*-GlcNAcylated on Ser733. In a p53-null context, it was associated with pro-oncogenic activity in a model of colitis-associated cancer. Second, NF-κB itself is also *O*-GlcNAcylated at Thr322 and Thr352 in presence of high glucose concentration. In pancreatic cancer cells, Thr322 and Thr352 *O*-GlcNAcylation were shown to be involved in cell anchorage *in vitro*. In breast and prostatic cancer cells, OGT controls the expression of FOXM1, a pro-oncogenic transcriptional factor. However, FOXM1, itself, is not *O*-GlcNAcylated. Through an unknown substrate and mechanism, OGT prevents FOXM1 degradation and thus promotes tumor development in breast cancer and metastasis in prostate cancer. β-Catenin, involved in cell adherent junctions, is also central to the Wnt/β-catenin pathway as a transcription factor in complex notably with LEF/TCF co-factors. Once activated, β-catenin promotes tumorigenesis, especially colorectal and liver cancers. Increased *O*-GlcNAcylation levels of β-catenin have been observed in colon carcinoma cells. Furthermore, interaction of OGT with β-catenin is stimulated during serum-induced proliferation in HeLa cells. These observations support a positive role of *O*-GlcNAc-modified β-catenin in cell proliferation. The well known tumor suppressor p53 is *O*-GlcNAcylated at Ser149 which prevents the phosphorylation at Thr155 by the COP9 signalosome. This results in an inhibition of p53 degradation. *O*-GlcNAcylation of p53 promotes p53 stability and thus its tumor suppressor activity in physiological context. In case of pro-oncogenic p53 mutants, *O*-GlcNAc-mediated stability of p53 may favor pro-oncogenic processes. This hypothesis has not been addressed experimentally yet.

Transcription factors bind DNA to control the expression of an array of target genes, consisting for some in genuine “hubs” for cellular process regulation. Mutation in transcription factor-encoding gene, resulting in dysregulation of their expression or activity, is a common mechanism involved in many cancers (88). Nonetheless, post-translational modifications including *O*-GlcNAcylation also represent an important control mechanism of the expression and/or activity of transcription factors (16, 89). Rightfully, several transcription factors involved in cancer have been shown to be *O*-GlcNAcylated (Figure 3).

### MYC

The protein MYC which is normally expressed at low levels is largely overexpressed in proliferative cells and cancer cells. MYC is a preponderant oncogenic transcriptional factor driving forward cell cycle and replication (90). Upon growth signals, phosphorylation on Ser62 results in stimulation of MYC activity. This is a prerequisite for GSK3β to phosphorylate MYC at Thr58 near the transactivation domain, resulting in its degradation (91). Almost 20 years ago, Thr58 was shown to be also *O*-GlcNAcylated (22, 92). GSK3β inhibition or mutation of Ser62 to Ala increase *O*-GlcNAcylation of Thr58. Interestingly, in lymphomas, Thr58 is frequently mutated resulting in a more stable MYC (92). Although it has not been clearly demonstrated that *O*-GlcNAcylation of Thr58 *per se* is able to stabilize MYC, it is reasonable to assume that in a context of hyper-*O*-GlcNAcylation, phosphorylation of MYC on Thr58 by GSK3β will be reduced, preventing its degradation. This would result in a more stable and more active MYC, promoting tumorigenesis. Although experimental evidences are still required to support such a mechanism, a very recent report presented data in line with this hypothesis (93). In prostate cancer biopsies, OGT is upregulated in association with MYC levels. In three prostate cancer cell lines (LNCaP, VCaP, and PC3), pharmacologically inhibition of OGT decreased MYC protein stability without affecting its mRNA levels. The authors confirmed *O*-GlcNAcylation of MYC in LNCaP cell line suggesting that this PTM could be responsible for MYC stabilization (93). However, to our knowledge, the *O*-GlcNAc status of MYC in patient tumors still remains to be evaluated.

### p53

p53 is a central tumor suppressor whose gene is mutated in more than 50% of mutations in cancers (94). p53 level is tightly controlled by PTM (95). Upon various stress signals (DNA damage, oncogenic events, hypoxia…), p53 levels are stabilized through inhibition of proteasomal degradation. This is achieved by phosphorylation of p53, which blocks its interaction with the E3 ubiquitin ligase MDM2 (95). However, phosphorylation-induced degradation of p53 can also occur, as illustrated by phosphorylation performed by COP9 signalosome on Thr155 in the DNA-binding region of p53 (96). More recently, Yang et al. showed that *O*-GlcNAcylation of p53 at residue Ser149 inhibits its phosphorylation on Thr155 by COP9 signalosome, hence promoting p53 stabilization and activity (97). In the case of a wild-type p53, *O*-GlcNAcylation should promote its tumor suppressor activity, as suggested by higher apoptosis in H1299 cells (97). However, examples of gain-of-function mutant of p53 favoring tumorigenesis have been described in the literature (98–100). In this context, stabilization-induced *O*-GlcNAcylation of gain-of-function mutant form of p53 may amplify its pro-oncogenic activity. Clearly, this hypothesis requires to be addressed experimentally.

### NF-κB

NF-κB transcription factors are involved in a wide variety of physiological and pathological processes including, immunity and inflammation, metabolism, and cancer (101, 102). In the basal state, NF-κB is sequestered by IκB in the cytosol. Canonical activation takes place by activation of IκB kinase complex which in turn phosphorylates and triggers IκB degradation. NF-kB is then released and enters the nucleus to activate its target genes (101). Originally, Yang et al. identified residues Thr322 and Thr352 as the sites of *O*-GlcNAc modification. However, only *O*-GlcNAcylation of Thr352 was central for transcriptional activation (103). *O*-GlcNAcylation of NF-κB in relation to cancer has not been directly addressed until recently. It was shown that mutation of Thr322 and Thr352 to Ala, reduced the anchorage of pancreatic ductal adenocarcinoma cells *in vitro* (104). Therefore, it will be of great interest to determine *in vivo* the involvement of NF-κB *O*-GlcNAcylation in the context of tumorigenesis.

Links between NF-κB pathway and *O*-GlcNAcylation have also been characterized upstream NF-κB factors, in relation to the activating kinase IKKβ. In a context of p53-null mutation, aerobic glycolysis is upregulated through an IKK-NF-κB pathway (105). It was shown that IKKβ is activated by phosphorylation concomitantly with an increase in its Ser733 *O*-GlcNAcylation (106). Ser733 is usually the target of an inhibitory auto-phosphorylation by the catalytic domain of activated IKKβ. *O*-GlcNAcylation of Ser733 prevents inhibition of IKKβ which results in a sustained activity. Consequently, NF-κB pathway remains active, and this further promotes glycolysis (106). The role of NF-κB pathway in tumorigenesis is complex, either pro- or anti-oncogenic, depending on cancer type, environment, inflammation status, or p53 functionality (105, 107–110). Whereas it now clearly appears that *O*-GlcNAcylation play a significant role in NF-κB pathway, its involvement in tumorigenesis still remains to be precisely defined.

### FOXM1

FOXM1 is a transcriptional regulator expressed at high level in various cancers, pointing out its importance in tumorigenesis (111). Links between FOXM1 and *O*-GlcNAcylation pathway have been made by Reginato’s laboratory (112). These authors showed that high levels of OGT are responsible for the increased activity of FOXM1 in breast cancer MCF-10A cells overexpressing the activated form of ErbB2. Although no *O*-GlcNAcylated form of FOXM1 could be detected, OGT expression was shown to be central to the control of FOXM1 levels and to its effects on cell proliferation (112). FOXM1 levels were dramatically decreased by OGT silencing. Consequently, the level of the FOXM1’s target S-phase kinase-associated protein 2 (Skp2), involved in the SCF^SKP2^ E3 ubiquitin ligase complex responsible for p27^Kip1^ degradation, was markedly decreased. This resulted in an increase in cell cycle inhibitor p27^Kip1^ protein levels and subsequent cell cycle arrest (112). Similarly, the same group showed that inhibition of OGT in the prostate cancer cell line PC3-ML was associated with an increase in FOXM1 degradation through a proteasome-mediated process (37). It was also observed that loss of FOXM1 upon modulation of OGT levels in PC3-ML cells affects their ability to form bone metastasis in mice (see below). Overexpression of a degradation-resistant mutant of FOXM1 rescued the effects of OGT inhibition (37). However, the direct target of OGT involved in the control of FOXM1 expression remains to be identified.

### β-Catenin

β-Catenin is a versatile protein playing fundamental roles in cells from control of intercellular junction integrity through its interaction with cadherins and cytoskeleton, to regulation of transcriptional processes as a co-transcription factor. As a cell cycle and proliferation regulator, β-catenin is a key partner in the Wnt/β-catenin pathway. Upon Wnt ligand interaction with its receptor, β-catenin sequestered by the Adenomatous Polyposis Coli (APC)/axin degradation complex is released and re-localized to the nucleus, where it interacts with TCF/LEF or other co-factors to form a transcriptional complex able to modulate the expression of a number of target genes (113). Mutations of β-catenin itself (accounting for 10% of mutations) or APC (one of the main component of the destruction complex, accounting for 80% of mutations), are found in 85–90% of CRC. These mutations increase β-catenin stability which therefore acquires oncogenic properties.

Recently, in an attempt to understand the link between nutrition and CRC, Olivier-Van Stichelen and colleagues showed that β-catenin expression correlated with the HBP flux and the *O*-GlcNAcylation levels in colon carcinoma cells (114) as previously pointed out in macrophages (115). Later, Olivier-Van Stichelen et al. showed that serum-induced proliferation increases the interaction between OGT and β-catenin in HeLa cells, supporting a positive role for *O*-GlcNAc-modified β-catenin in cell cycle progression (27). It is therefore proposed that hyper-*O*-GlcNAcylation in the colic and rectal mucosa may constitute a pro-oncogenic signal, explaining why metabolic disorders and over-nutrition increase the risk of CRC.

### Other transcription factors

Obviously, other transcription factors involved in cancer have been shown to be *O*-GlcNAc-modified as hypermethylated in cancer 1 (HIC1) (24), Jun (116, 117), Estrogen Receptor β (118, 119), the chimeric transcription factor EWS-FLI1 expressed in Ewing’s sarcoma family tumors (120). However, significant work remains to be performed to better understand the exact role of the *O*-GlcNAc-modified residues of oncogenic-related transcription factors and their function in tumorigenesis.

## *O*-GlcNAcylation Regulates Cell Adhesion and Migration

Numerous studies indicate that *O*-GlcNAc promotes cancer cell invasiveness and metastasis. Thus, manipulation of *O*-GlcNAc levels using chemical or shRNA-mediated inhibition of OGA or OGT affects *in vitro* and *in vivo* migration/invasion of breast (31, 112, 121), lung (31, 33), liver (34), prostate (37), and colon (33) cancer cells. Several lines of evidence suggest that *O*-GlcNAc favors cancer malignancy by impacting the E-cadherine/β-catenin system and by promoting expression of metalloproteinases.

### E-cadherin and cancer metastasis

E-cadherin, the prototypic member of the cadherin family, is a major component of adherent junctions. E-cadherin regulates cell–cell adhesion via homophilic interactions between its extracellular domains on opposing plasma membranes and by binding of its intracellular domain to the cytoskeleton via β-catenin. Down-regulation of E-cadherin is a critical step in the epithelial-mesenchymal transition (EMT), characteristic of carcinoma invasion. In breast cancer 4T1 cells, E-cadherin protein expression was increased by OGT shRNA, while it was decreased by treatment with the pharmacological OGA inhibitors PUGNAc and NButGT (31). Moreover, co-immunoprecipitation experiments showed that E-cadherin interaction with β-catenin and p120 was markedly increased in OGT depleted cells and decreased by OGA inhibitors (31). In addition, analysis of Triton-X100 soluble and insoluble fraction showed that association of E-cadherin, β-catenin, and p120 with cytoskeleton was increased by shOGT and decreased in OGA inhibited cells. Immunofluorescence experiments indicated that shOGT increased colocalization of E-cadherin, β-catenin, and p120 at the cell surface, whereas OGA inhibition reduced it. *In vivo* 4T1 cells metastasis in the lung was markedly reduced by OGT shRNA, and inhibition of E-cadherin expression in these cells (shOGT + shCadherin) restored invasiveness to control levels, suggesting an important role for regulation of E-cadherin expression by *O*-GlcNAc in cancer cell invasiveness (31). Whereas no change in E-cadherin *O*-GlcNAcylation was reported in this study, *O*-GlcNAc could be detected on p120 and β-catenin, suggesting a potential mechanism for *O*-GlcNAc-induced inhibition of E-cadherin cell surface localization. In addition, works from other laboratories suggested that *O*-GlcNAcylation regulates E-cadherin expression at the transcriptional level (34, 121). Indeed, increasing *O*-GlcNAcylation through siRNA-mediated inhibition of OGA down-regulated E-cadherin mRNA expression in LC cells, whereas decreasing OGT expression had the opposite effect (34). Interestingly, Snail1, a key regulator of EMT program, and a major transcriptional repressor of E-cadherin, was is *O*-GlcNAcylated in hyperglycemic conditions in an OGT-dependent manner (121). GSK3-mediated phosphorylation and degradation of Snail1 is suppressed by its *O*-GlcNAcylation. Stabilization of Snail1 increases its repressor function, resulting in inhibition of E-cadherin mRNA expression (121). Therefore, *O*-GlcNAc regulation decreases E-cadherin localization at the membrane through inhibition of its interaction with its partners p120 and β-catenin, but also through stabilization of its transcriptional repressor Snail1. Consistently, a recent study showed a positive correlation between *O*-GlcNAc protein levels, cell migration, and E-cadherin protein levels in ovarian cancer cells. In HO-8910PM cells, down-regulation of OGT resulted in an increase in E-cadherin protein content. On the opposite, increased *O*-GlcNAcylation level through PUGNAc or Thiamet-G treatment decreased E-cadherin protein content in OVCAR3 cells (122). The authors showed that increased *O*-GlcNAcylation inhibits E-cadherin-catenin complex formation and that both E-cadherin, p120 and β-catenin can be *O*-GlcNAcylated in these cells (122). Identification of the *O*-GlcNAc-modified sites in the different partners involved will be required to precisely understand the mechanism involving *O*-GlcNAcylation in E-cadherin-dependent cancer cell migration.

### Increased expression of matrix metalloproteinases

Matrix metalloproteinases (MMPs) are zinc-finger dependent extracellular matrix (ECM) remodeling enzymes. Overexpression of MMPs has been associated with epithelial to mesenchymal transition. MMPs play crucial roles in invasion and metastasis, through proteolytic degradation of ECM, alteration of cell–cell and cell-ECM interactions, migration, and angiogenesis. The proteolytic activity of MMPs is required for a cancer cell to degrade physical barriers during local expansion, intravasation at nearby blood vessels, as well as extravasation and invasion at a distant location (123). Reginato’s group first demonstrated negative regulation of MMP2 expression upon OGT inhibition using OGT shRNA in breast cancer cells (an effect probably mediated by down-regulation of FOXM1 protein), associated with decreased cell invasiveness (112). In LCs cells, down-regulation of OGT expression using siRNA resulted in decreased expression of MMP1, MMP2, and MMP3, correlated with the decreased migrating and invasive capabilities of these cells, whereas down-regulation of OGA had the opposite effects (34). More recently, the Reginato’s group showed that reducing *O*-GlcNAc in prostate cancer cells also reduced the expression of MMP2 and MMP9, associated with decreased FOXM1 expression (37). Inhibition of OGT expression in these cells was also associated with decreased *in vitro* invasiveness and *in vivo* metastasis. Interestingly, VEGF expression and angiogenesis were also inhibited by OGT siRNA, further emphasizing the involvement of *O*-GlcNAcylation in malignant properties of the cells. *O*-GlcNAcylation then participates in an integrative strategy that promotes invasiveness and metastasis by increasing ECM degradation in order to clear the way for migration.

## Conclusion and Future Directions

Our knowledge regarding the regulation of cell cycle by *O*-GlcNAcylation progresses at a relatively high rate. Conversely, apprehending the role of *O*-GlcNAcylation in cancer processes is only at its beginnings. As reviewed here, there are many proofs that *O*-GlcNAcylation processes are perturbed in different kinds of cancers. Mainly, it appeared that *O*-GlcNAcylation and its cycling enzymes are upregulated in cancers and that this elevation is positively correlated with the grade or the aggressiveness of the tumor. It also appeared that metastases harbor higher *O*-GlcNAcylation levels than the primary tumor. Nevertheless, as mentioned previously, a number of contradictions concerning the expression or activity of *O*-GlcNAc-cycling enzymes also appeared (30–36, 40, 46). The large variety of different types of cancers and cellular models used in these studies, and the fact that *O*-GlcNAc level may itself modulate the expression of cycling enzymes deeply complicates the interpretation of the data. Future development should certainly include detailed analysis of the regulation of the promoters of OGT and OGA, and the study of the transcription factors that control the activities of these promoters in different types of normal and cancer cells.

The lack of tools necessary to specifically study the impact of *O*-GlcNAcylation on carcinogenesis may also partly explain contradictions observed in literature and slow down the progression toward understanding whether and how *O*-GlcNAc and its cycling enzymes influence the development of cancers. A striking feature of *O*-GlcNAcylation in cancer is the variety of proteins targeted by this PTM. In addition to cell cycle proteins, signaling intermediates and transcription factors already discussed in this review, several other types proteins have been shown to be *O*-GlcNAcylated in cancer, including metabolic enzymes (124) and heat shock protein (125, 126). From this impressive diversity, the fact that only two enzymes, OGT and OGA, are the only catalytic proteins in charge, calls for further identification of their co-factors and targets and for a better understanding of their mutual interaction, as recently exemplified with TET2/3 and OGT in epigenetic control (85, 87).

Modulating *O*-GlcNAc pathway could constitute a promising approach for anti-cancer therapy, which could be used in synergy with conventional treatments. This will be possible with systematic identification of *O*-GlcNAc-modified sites on the proteins of interest, which remains the prerequisite to understand the role of this PTM in their function. Our understanding in *O*-GlcNAc proteome is rising exponentially (20). However, strategies to alter *O*-GlcNAc-cycling enzymes and their targets in order to specifically block cancer-related processes still need to be developed. The important number of cancer types renders difficult to predict how conserved a mechanism would be from one tumor to the other. A way to tackle this issue may come from the determination of the *O*-GlcNAcylated protein signature in the different cancers. This clearly remains a wide field for investigation. Although pharmacological targeting of OGT is possible *in vitro* (127), important work is still required for *in vivo* application. Indeed, keeping in mind that *O*-GlcNAcylation in normal cells should remain untouched, targeting OGT specifically in cancer cell *in vivo* may represent one of the most important challenges in this field. *O*-GlcNAc-modulating strategies will indeed require caution, as increasing amount of evidences suggests that *O*-GlcNAcylation regulates highly important biological processes such as epigenetic (77) and affects molecular mechanisms involved in major human diseases such as Alzheimer’s disease, cardiovascular disorders, and diabetes (17, 18).

As we have seen throughout this review, considerable amount of evidence indicates that *O*-GlcNAcylation plays an important role in cell in cancer, through a broad panel of mechanisms (cell cycle, chromatin dynamics, transcription factors, kinases, and phosphatases involved in cell signaling, cell adhesion…). However, it is not clear whether cancer development requires increase in global protein *O*-GlcNAcylation or only modification of specific key proteins. In addition, as *O*-GlcNAcylation level tightly depends on nutrient availability, an important challenge will be to establish, for each protein identified as modified by *O*-GlcNAc in cancer cells, whether this modification is a cause or a consequence of the cancerous phenotype of the cell. Indeed, metabolic reprograming, one of the so-called cancer hallmarks, include increased glucose and glutamine uptake, which might be sufficient to increase *O*-GlcNAc level in proteins. This may promote a general increase in *O*-GlcNAc level on proteins, including in proteins more specifically involved in the maintenance of the cancerous phenotype, leaving the question of causality difficult to answer.

Finally, because the analogy between *O*-GlcNAcylation and phosphorylation is striking, we are always tempted to apply to *O*-GlcNAc signaling reasoning schemes and concepts taken from our classical view of phosphorylation cascades and their alterations in cancer cells. However, important differences should be kept in mind. Unlike phosphorylation, *O*-GlcNAcylation, which is performed by a single enzyme, cannot organize in signaling cascades in which one *O*-GlcNAcylated protein will transmit the signal by *O*-GlcNAcylating another protein. Rather than acting as a switch that turns on or off signaling pathways, *O*-GlcNAcylation should be considered as a “rheostat” (17) that controls the intensity of signals traveling through various pathways according to nutrient and stress environment. Whether one of the ancestral functions selected by evolution in unicellular organisms was to use this “rheostat” to sense nutrient availability and promote proliferation when the nutritional environment was favorable remains an open question that might provide some clues to understand why *O*-GlcNAcylation appears to be central to cancer cell biology.

## Conflict of Interest Statement

The authors declare that the research was conducted in the absence of any commercial or financial relationships that could be construed as a potential conflict of interest.
